# PD-L1 Expression on Circulating Tumour Cells May Be Predictive of Response to Regorafenib in Patients Diagnosed with Chemorefractory Metastatic Colorectal Cancer

**DOI:** 10.3390/ijms21186907

**Published:** 2020-09-20

**Authors:** Lucrezia Raimondi, Filippo Maria Raimondi, Laura Di Benedetto, Giuseppe Cimino, Gian Paolo Spinelli

**Affiliations:** 1 UOC Territorial Oncology of Aprilia, AUSL Latina, University of Rome Sapienza, 04011 Aprilia, Italy; lucrezia.raimondi@uniroma1.it (L.R.); giuseppe.cimino@uniroma1.it (G.C.); 2 Biostatistical Consultant, 00197 Rome, Italy; filippo3006@gmail.com; 3 BIOS SpA, 00197 Rome, Italy; lauradibenedetto.ldb@gmail.com

**Keywords:** chemorefractory metastatic colorectal cancer, regorafenib, circulating tumour cells, PD-L1, PLR, liquid biopsy, acquired resistance, targeted therapy

## Abstract

Regorafenib, targeting a broad range of receptor tyrosine kinases (RTKs), is an oral multikinase inhibitor which improves the progression-free survival (PFS) and overall survival (OS) of patients diagnosed with chemorefractory metastatic colorectal cancer (mCRC), making an immunosuppressive tumour microenvironment. The correlation between PD-1/PD-L1 expression and RTKs inhibition has been studied in several tumour types but has not been analyzed extensively in mCRC in the era of regorafenib. In this study, using liquid biopsy, we evaluated the opportunity to reveal if PD-L1 expression on circulating tumour cells (CTCs) could serve as a predictive biomarker of response and clinical benefit in patients treated with regorafenib as the third line of treatment. We analyzed a cohort of forty chemorefractory metastatic colorectal cancer patients, of whom twenty-six *KRAS* mutated, treated with regorafenib, all as the third line of treatment. Blood samples were collected from patients prior to treatment and longitudinally four and eight weeks after initiation of therapy. CTCs were identified using multiparametric flow cytometry; therefore, PD-L1 expression was evaluated. Objective responses were defined following the RECIST criteria v.1.1. Moreover, focusing on peripheral blood biomarkers, we found that high platelet-to-lymphocyte ratio (PLR) was an independent prognostic indicator of poor OS. For the first time, our study showed the usefulness of sequential assessments of CTCs as a non-invasive real-time biopsy to evaluate PD-L1 expression in patients diagnosed with mCRC and treated with regorafenib. Our analysis suggests that by assessing PD-L1 expression on CTCs, we could predict who will benefit from regorafenib, offering highly individualized treatment plans.

## 1. Introduction

Colorectal cancer (CRC) is the third and second most frequent cancer in men and women, respectively, and with 694,000 deaths/year worldwide is one of the leading causes of cancer-related deaths, with an incidence of about 9%, estimated yearly [1,2].

Despite progress made in the setting of early diagnosis and treatment, a large number of patients are diagnosed in the advanced disease stage, presenting distant metastases from the diagnosis. In addition, half of the patients who undergo surgery will develop metastases because of circulating tumour cells (CTCs), and tumour-derived exosomes (TDE) in some cases [3].

Chemotherapy, with Fluoropyrimidine, Oxaliplatin, or Irinotecan given sequentially or in combination, is considered the backbone of management for metastatic colorectal cancer (mCRC), even if monoclonal antibodies targeting vascular endothelial growth factor (VEGF) and epidermal growth factor receptors (EGFR) in *RAS* wild-type tumours provided an additional mechanism for disease control in this cohort of patients [4]. Recently, the survival outcomes have improved, although modestly, with the introduction of a new agent.

Regorafenib, an oral multi-kinase inhibitor, was approved as third line therapy for mCRC previously treated with chemotherapy, regardless of previous anti-VEGF or anti-EGFR therapy, based on the results of the phase III CORRECT trial. Regorafenib has been shown to block the activity of multiple protein kinases active in tumour angiogenesis, oncogenesis and in the modulation of the tumour microenvironment [5,6].

Only a small portion of patients diagnosed with mCRC benefits from regorafenib and, taking into consideration the side effect profile and the high cost of the drug, biomarkers identifying which patients could benefit from this drug are just as crucial as they are lacking.

Compared to tissue biopsy, which is the current gold standard for mCRC diagnosis and prognosis, circulating tumour cells (CTCs) hold promise to better reflect the tumour heterogeneity, providing complementary information about the whole tumour [7]. In addition, they can be analyzed longitudinally as liquid biopsies, a non-invasive tool that provides real-time data on changes in tumors [8].

Specific detection of CTCs in cancer patients remains a challenge. The Food and Drug Administration (FDA) approved the semiautomated, EpCAM/pankeratins(K)-dependent CellSearch^®^ system as a CTC detection method, but its usefulness in mCRC remains challenging.

PD-L1 expression is the most studied biomarker in immune-oncology, especially in non-small cell lung cancer (NSCLC), for its predictive value [9]. However, a growing number of studies have demonstrated a higher response to chemo-immunotherapy in patients with high PD-L1 expression in their NSCLC tumors [10,11].

To the best of our knowledge, this is the first study to assess PD-L1 on CTCs using the CellSearch^®^ system in a cohort of patients diagnosed with mCRC in treatment with regorafenib as the third line of treatment. Furthermore, we evaluated the opportunity to reveal if PD-L1 expression on CTCs could serve as a predictive biomarker of response and clinical benefit in patients treated with regorafenib.

## 2. Results

### 2.1. Patient Characteristics and PD-L1 Detection on CTCs

Our study identified a total of 40 consecutive patients, all of them Caucasian, diagnosed with chemorefractory metastatic colorectal cancer. Treated with standard-of-care regorafenib as third-line metastatic therapy, they were enrolled in the study between September 2019 and June 2020 and they were assessable for the analysis of PD-L1 CTCs and PLR.

Patients who did not complete blood draw were excluded from the analysis.

The main baseline clinicopathological characteristics of the 40 patients are presented in Table A1 (Appendix A).

All patients were assessable for the analysis and a total of 120 blood samples were collected at baseline, before starting regorafenib treatment, and after 4 and 8 weeks of treatment. CTCs were detected in 38 of 40 patients (95%), ranging from 12 to 410 cells in 7.5 mL of blood. CTCs were significantly associated with the presence of *RAS* mutation.

**Figure 1:**
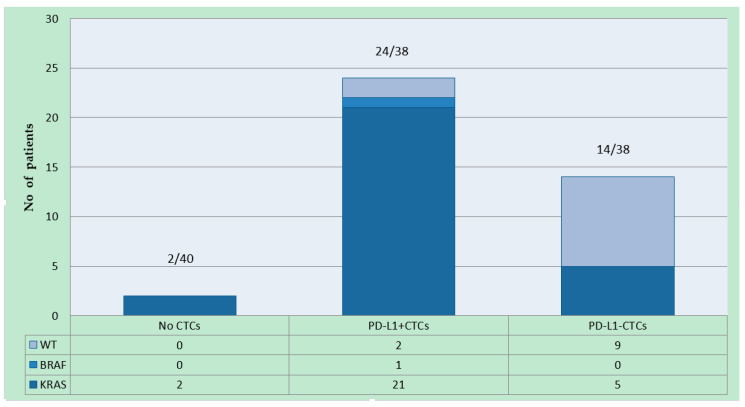
CTCs and PD-L1 expression on CTCs detected. Number of patients with only PD-L1^−^, only PD-L1^+^ is reported in the chart below, in relation to *KRAS* and *BRAF* mutational status.

*KRAS* mutations were previously detected in 26 patients (65%). Twenty-two had a mutation in codon G12D and G13D (22/26, 84.6%). Less common *KRAS* mutations were found in three patients (i.e., G12A in two patients and A146T in another one); in one patient, two mutations were found (i.e., G12D/G12V). A mutation in codon V600E of the *BRAF* gene was detected in the ctDNA of a patient. CTCs were detected in all patients with both *KRAS* and *BRAF* mutations (Figure 1).

**Figure 2:**
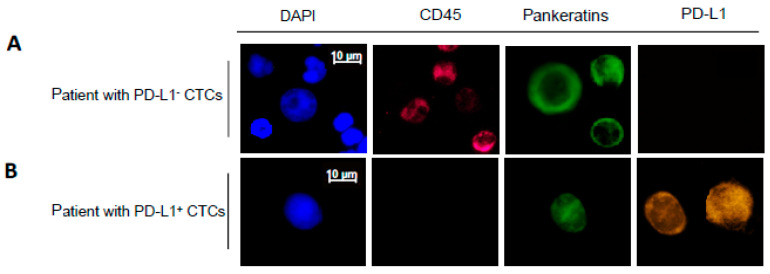

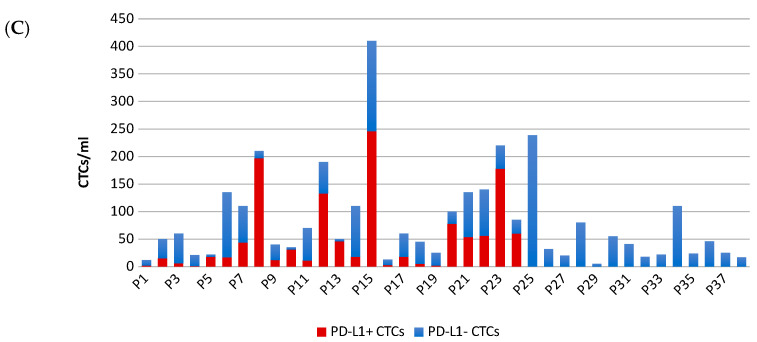
Representative images of CTCs detected and subjected to immunostaining with DAPI, CD45, Pankeratins and PD-L1. Example images of CTCs from a patient with PD-L1^−^ CTCs (**A**) and PD-L1^+^ CTCs (**B**) are shown. The scale bar of 10 μm was applied to all pictures. (**C**) Number of CTCs and PD-L1 status isolated from blood samples from 38 patients (P1 through P38) with detectable PD-L1 status. “P1” stands for patient 1. Red bar represents the number of CK(+)/PD-L1(+) CTCs. Blue bar represents the number of CK(+)/PD-L1(−) CTCs.

PD-L1 was identified in 24 of the 38 patients (24/38, 63.2%) in whom CTCs were detected at baseline. The percentage of CTCs expressing PD-L1 varied widely, ranging between 5 and 94%. Of 24 patients with PD-L1^+^ CTCs, and 21 (21/24, 87.5%) with *KRAS* mutation, one patient (1/24, 4.2%) was identified with *BRAF* mutation and two (2/24, 8.3%) patients without mutations were identified. Fourteen patients, 5 (5/14, 35.7%) of whom had *KRAS* mutation, had PD-L1^−^ CTCs (Figure 2).

### 2.2. Patient Characteristics and PD-L1 Detection on CTCs

**Figure 3:**
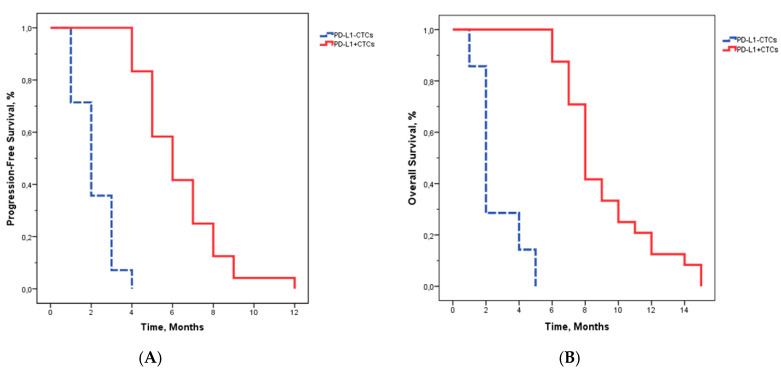
Kaplan–Meier plots of progression-free survival and overall survival according to PD-L1 expression on CTCs prior to regorafenib. (**A**): Progression-free survival. (**B**): Overall survival.

A statistically significant difference in PFS was observed between patients with PD-L1^+^ CTCs compared with patients with PD-L1^−^ CTCs (*p* < 0.001, with a hazard ratio of 0.182; 95% CI 0.051–0.594). The presence of PD-L1^−^ CTCs was significantly associated with worse PFS (median PFS: 2.1 months, range 1–4 months). The median PFS for PD-L1^+^ CTCs patients was 6.3 months, range 4–12 months). PD-L1^−^ CTCs group had a median OS of 2.5 months (range 1–5 months) whereas the median OS was 9.1 months (range 6–15 months) for the group with PD-L1^+^ CTCs (Figure 3). Further information in Table A2 (Appendix B).

We prospectively collected longitudinal samples of patients undergoing therapy with regorafenib and objective responses were defined following the RECIST criteria v.1.1. Collecting a pre-treatment sample, one sample at the time of first staging (after 4 weeks of treatment), and one sample upon progression, we quantified PD-L1^+^ and PD-L1^−^ CTCs at each time point. This strategy allowed us to assess the dynamics of CTC counts and of the fraction of PD-L1^+^ CTCs upon regorafenib.

We divided patients into two groups in relation to clinical benefit achieved: on the one hand, patients who achieved partial remission or stable disease (no patients achieved complete remission), on the other those with progressive disease at the first staging or after an initial response.

Two months after starting treatment, the objective response rate (ORR) among all patients enrolled was 5.3% and the clinical benefit rate (CBR) was 55.3%. Among patients with CTCs detectable, two (5%) patients achieved a partial response (PR), 19 (50%) stable disease (SD) and 17 (45%) had a progressive disease (PD).

We observed that patients with partial response or stability of disease had PD-L1^+^ CTCs whereas patients with PD-L1^−^ CTCs had a progressive disease (*p* < 0.001). PD-L1 status provided critical information for the prediction of therapeutic responses. At 12-month follow-up, the median duration of response (DOR) was 2 months for the PD-L1^−^ CTCs group and 7 months for the PD-L1^+^ CTCs group (*p* < 0.001). The number of PD-L1^+^ CTCs was significantly higher in responders (*p* = 0.002)

**Figure 4:**
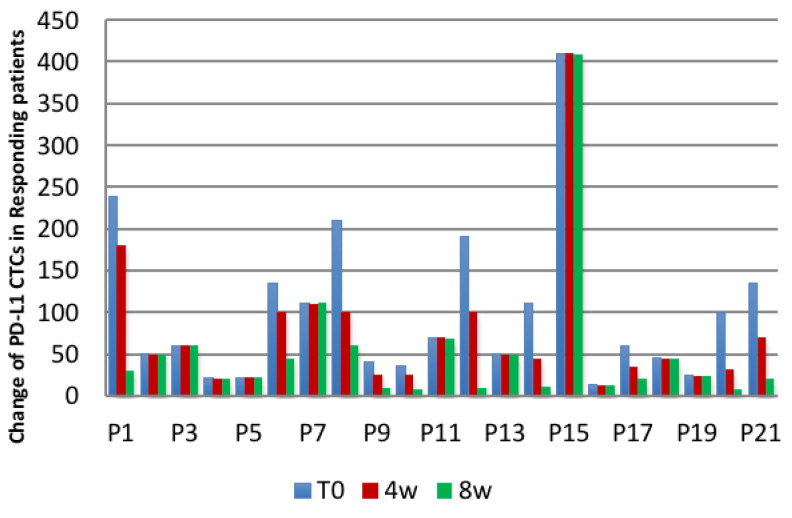

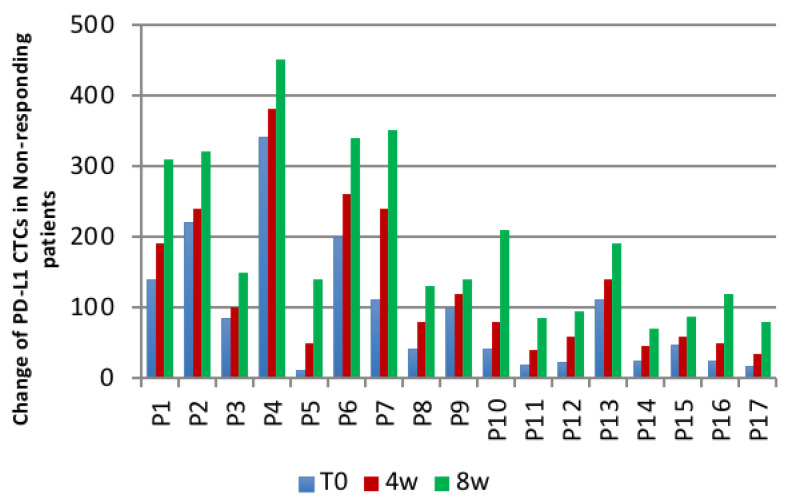
We showed the changes in PD-L1^+/−^ CTCs in relation to the clinical response to regorafenib. Two patients with partial remission and 19 with stable disease had a decrease/no change in PD-L1 CTCs compared to 17 patients with progressive disease, who had an increase in PD-L1^−^ CTCs to initial diagnosis. T0: Baseline; 4W: Assessment 4 weeks later; 8W: Assessment 8 weeks later.

With a cut-off of at least one PD-L1^+^ CTCs, a receiver operating characteristic (ROC) curve for prediction of response had 75.1% sensitivity with 91.1% specificity. Comparing CTCs detected at baseline and after 4 and 8 weeks of starting treatment, increased CTC levels were linked to poor prognosis and progressive disease (*p* = 0.002), meanwhile a decrease in CTC number was significantly associated with response to treatment (*p* = 0.014). All responding patients (21/21) showed a decrease (10/21) or no change (11/21) in PD-L1^+/−^ CTCs at the time of response compared to before initiation of regorafenib. On the other hand, all patients showed an increase in PD-L1^−^ As illustrated in Figure 4, CTCs at progression compared to either initiation of treatment (*p* = 0.001, decrease or no change vs. increase of CTCs at response vs. Progression, Fisher’s Exact test) (Table 1).

### 2.3. Relationship between Platelet-To-Lymphocyte Ratio (PLR) and Patients’ Outcomes

**Figure 5:**
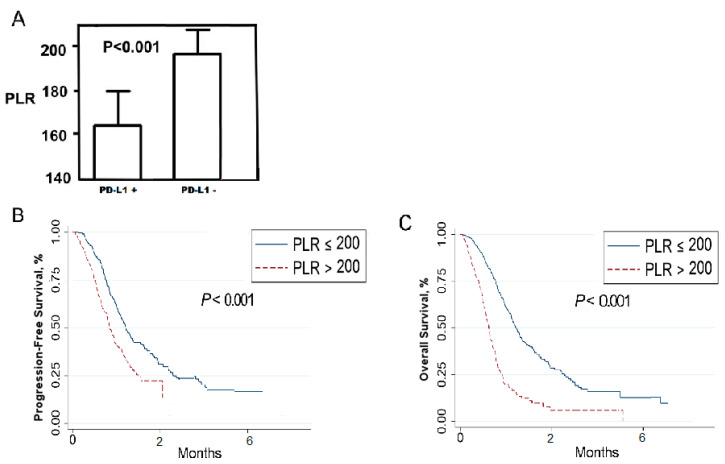
Relationship between PLR and PD-L1^+/−^ CTCs (**A**): patients with PD-L1+ CTCs had lower PLR. Patients treated with regorafenib in the PLR-low group had a better PFS (**B**) and OS (**C**) compared to patients in the PLR-high group.

The median value of PLR was 212.51 ± 208.32 (mean ± standard deviation) with a hazard ratio of 5.11 (*p* < 0.001, 95% CI 1.945–14.121). A time-dependent ROC curve was performed and the area under the curve (AUC) was 98.6%. Overall, sensitivity and specificity were 95.6% and 98.9%. When the cut-off was set to 200, a significant difference in PLR was found between patients who developed relapse (median 268.6, range 185.0–533.3) and those who did not develop relapse (median 131.8, range 32.1–240.2) (*p* = 0.03). Patients treated with regorafenib in the PLR-low group had a better PFS and OS (*p* < 0.001) (Figure 5). No other significant association was found comparing the baseline levels of white blood count and lymphocytes in the overall population with respect to the type of response and PFS (*p* ≥ 0.05) (Table 2).

## 3. Discussion

Metastasis is responsible for 90% of cancer-related death: better understandings of the biology that govern this process are required and still under investigation. Metastatic cells, moving from the primary tumour, invade and colonize a distant tissue, growing until they form macroscopic metastatic lesions. Epithelial-mesenchymal plasticity has been considered as a key event for tumour dissemination for the loss of polarity and cell–cell adhesions [12].

Circulating tumour cells (CTCs), acquiring key properties required for metastatic spread and circulating as single or clusters of cells, may be considered as an intermediate stage of metastasis [13,14]. CTCs, providing information on dynamic changes in tumour burden, are considered to be potential real-time liquid biopsies useful for selection of the target therapy [15].

While the potential application of immune gene signature and tumour-infiltrating lymphocytes has been increasingly reported, the clinical utility of the expression of PD-L1 in CTCs has been so far the subject of limited investigation. To date, only few studies have analyzed the PD-L1 expression in CTCs of patients diagnosed with breast cancer, NSCLC, head/neck cancer, prostate and bladder cancer and melanoma [16,17,18,19,20,21]. PD-L1 expression in tumour biopsies has been shown to be a predictive biomarker for the efficacy of immune checkpoint inhibitors and for patients’ clinical outcomes [22]. Nevertheless, because of intra-tumour heterogeneity and clonal evolution that make tissue sampling a suboptimal portrayal of the tumour molecular profile, PD-L1 expression on CTCs could be by far more convenient for both clinicians and patients, allowing a longitudinal assessment of PD-L1 expression during the different clinical phases of the disease [23].

In the literature, there are very few data about the expression of PD-L1 on CTCs in patients diagnosed with NSCLC and melanoma but, to the best of our knowledge, our study is the first to analyze PD-L1 expression on CTCs isolated from patients with chemorefractory mCRC in the era of regorafenib.

Our study indicated the presence of PD-L1 CTCs in patients with treated mCRC. In almost all patients enrolled (38/40, 95%), CTCs were detected. Our results indicate better and longer response rates in patients with PD-L1^+^ CTCs at baseline and in those in whom their number was decreased 4 and 8 weeks after initiation of regorafenib, compared to the time point before starting. Instead, the increase in PD-L1^+^ CTCs is associated with progressive disease and a shorter response rate: seventeen out of seventeen patients with progressive disease and a shorter PFS and OS, had PD-L1^+^ CTCs, whereas none of the responding patients had an increase in PD-L1^+^ CTCs (10 patients had a decrease and 11 had no change in PD-L1^+^ CTCs). An increase in PD-L1^+^ CTCs after one or more months of treatment with regorafenib suggests that PD-L1^+^ tumour cells may be resistant to this therapy, resulting in progressive disease.

Thus, longitudinal blood collection and PD-L1 on CTCs analysis may be a useful tool to differentiate between responders and non-responders. A recent study including 35 patients diagnosed with different gastrointestinal tumours revealed that the majority of patients with an increase in PD-L1 CTCs had progressive disease [24]. The reason why an increase in PD-L1^+^ CTCs is linked to progressive disease is currently not understood: a hypothesis of recent formulation asserts that PD-L1^+^ CTCs represent more aggressive CTCs with an EMT-like phenotype [25,26,27].

The detection of CTCs was associated with patient’s clinical outcome: at baseline, the detection of PD-L1^−^ CTCs was associated with shorter PFS and OS compared with patients with PD-L1^+^ CTCs. It is likely that PD-L1 expression on CTCs may be involved in an interactive crosstalk between CTCs and immune cells, even if future works with larger cohorts of patients are needed to address this point.

Moreover, in the present study we demonstrated that the PFS and OS of patients with low PLR at baseline was significantly better than that of patients with a high PLR. As identified by Virchow, inflammation and malignancies are related [28]. Platelets play an important role in the development and especially progression of malignancies, even if the association between thrombocytosis and malignancies has not been clarified yet. Further, lymphocyte count reflects patients’ immune status and a potential immunity against cancer cells: a high PLR may indicate low immunity against the tumour. Elevated PLR may have a close association with worse survival in mCRC patients. In light of this, PLR could serve not only as a prognostic, but also as a predictive indicator of the immune state in the tumour microenvironment, allowing the identification of those patients with worse prognosis.

It is, however, necessary that further studies with larger cohorts, better if in the context of randomized clinical trials, be carried out to investigate both the prognostic and predictive value of PD-L1^+^ CTCs, in order to move the PD-L1 status of CTCs forward as a potential biomarker in chemorefractory mCRC in the era of regorafenib.

## 4. Materials and Methods

### 4.1. Patients, Treatment and Blood Collection

Forty patients diagnosed with mCRC were enrolled between June 2019 and July 2020. All patients gave written informed consent. This study has been conducted according to the Declaration of Helsinki.

After enrolment, a total of 120 pairs of blood samples were collected from patients with histologically proven mCRC. From each patient, at least 7.5 mL of whole blood was drawn with standard procedure peripheral vein blood, collected into ethylenediaminetetraacetic acid (EDTA) tubes (Sarsted, Nürnbrecht, Germany) and/or CellSave tubes (Menarini Silicon Biosystems, Florence, Italy) and stained immediately for flow cytometry analysis. The first 2 mL of blood were discarded to prevent contaminations by skin epithelial cells. At the time of sample collection, all patients had metastatic disease (100%, *n* = 40) and all of them were previously treated with at least two different lines of chemotherapy. Longitudinal samples were collected before starting regorafenib, after four and eight weeks, and at the time of progression from treatment. The PD-L1 status was assessed in tissue biopsies (PD-L1 tumour proportional score, TPS) and compared with the PD-L1 status of CTCs.

### 4.2. Detection and Identification of CTCs

The CTC enumeration was carried out through the CellSearch^®^ system, employing the CellSearch^®^ Epithelial Cell Kit (Janssen Diagnostics, LLC, Raritan, NY, USA), a semiautomated EpCAM/pankeratin-based CTC enrichment technique. It enumerates tumour cells of epithelial origin (CD45−, EpCAM+ and keratins 8, 18, and 19) in whole blood. The immunofluorescent technique was performed to identify the number of isolated CTCs and tumour cells from the blood sample. After fixation using 2% paraformaldehyde (PFA), cells were stained on a glass slide using, sequentially, 40,6-diamidino-2-phenylindole (DAPI, Janssen Diagnostics, Raritan, NJ, USA; 1:1000), pankeratins (AE1/AE3, 53-9003-82, eBioscience, Waltham, MA, USA; and C11, #4523, Cell Signaling Technology, CST, San Diego, CA, USA; 1:300 each), CD45 (a hematopoietic white blood cell marker, HI30, # 304018, BioLegend, Beverly, MA, USA, 1:200) and PD-L1 (D84TX, #86744, CST, San Diego, CA, USA; 1:50). The cells were subsequently washed with phosphate buffered saline and examined by fluorescence microscopy at 40× magnification. A cell was identified as a CTC if it demonstrated positive staining for EpCAM, K, DAPI and negative stains for CD45 and CD34. The results were quantitatively reported as the number of CTCs per 7.5 mL of whole blood.

The PD-L1 staining was established using spiked human blood samples. A Ficoll gradient was performed from 7.5 mL blood samples taken from healthy individuals at the Transfusionsmedizin (UKE, Germany). The mononucleated layer was collected and subsequently spiked with MCF7 cells (K+/PD-L1^−^) or H1975 cells (K+/PD-L1^+^). The spiked samples were spun on a glass slide by centrifugation. The generated slides were stained as described above to develop a specific staining protocol. Complete absence of staining was regarded as negative for PD-L1.

### 4.3. Treatment Response and Disease Progression Assessment

Tumor responses were assessed radiologically by computed tomography (CT) and positron electron tomography (PET) scans every two months. Response to treatment was defined on the basis of individual CT or PET scan.

### 4.4. Measurements of Platelet to Lymphocyte Ratio (PLR)

At baseline, the same day just before the start of regorafenib treatment and every four weeks of treatment (the same day just before the start of the following cycle), blood samples for complete blood count (CBC) were taken from the antecubital vein into the tubes containing potassium EDTA. Platelet and lymphocyte counts were determined automatically with haematology analyzer. Platelet to Lymphocyte ratio (PLR) was obtained for each patient by the absolute platelet count (cells/mm^3^) divided by the absolute lymphocyte count (cells/mm^3^) derived from the complete blood count of the patient. Using 150 as cut-off value, we divided patients into different groups: PLR-low and PLR-high.

### 4.5. Data Analysis

Patients were divided into those with at least one PD-L1 positive CTC (CTC PD-L1^+^) and those with PD-L1 negative CTC (CTC PD-L1^−^). Continuous variables are presented as mean ± SD or median ± IQR, depending on the shape of the distribution curve. Categorical variables are summarized with counts and percentages and were compared by Χ2 or Fisher’s exact tests. The correlation between CTCs detection and clinicopathological variables was examined using the nonparametric Mann–Whitney test for numerical data and the chi-square test for categorical data. We used Fisher’s exact test for the comparison of change of PD-L1^+^ CTCs in responding vs. non-responding patients during treatment with regorafenib.

Kaplan–Meier curves were generated and assessed using the log-rank (Mantel–Cox) test. For multivariate survival analysis, all variables were included in a forward logistic regression analysis. In the multivariate analysis, all variables with significance in the univariate analysis were included. Results are presented as hazard ratio (HR) with 95% CI and *p*-values. The optimal cut-off values as well as sensitivity and specificity were determined according to the receiver operator characteristic (ROC) analysis. The best cut-off values were expressed using the Youden index. The area under the ROC curve was calculated. Statistical significance was defined as *p* < 0.05. Statistical analysis was performed using SPSS Statistics 20.0 (IBM, Armonk, NY, USA).

## 5. Conclusions

In conclusion, we demonstrated that the determination of the PD-L1 status is feasible in the CTCs of chemorefractory mCRC patients. Patients with PD-L1^+^ CTCs had a higher response rate to regorafenib as well as longer PFS and OS. Hence, our study has showed the usefulness of detection of PD-L1^+^ CTCs at baseline and longitudinally with non-invasive real-time liquid biopsies to select those patients diagnosed with mCRC who could benefit from treatment with regorafenib, allowing us to offer ever-more highly individualized treatment plans.

## Figures and Tables

**Table 1:** Changes in PD-L1 ^+/−^ CTCs in relation to the clinical response to regorafenib. CTCs detected at baseline and after starting treatment.

Patients	Number of Patients with a	
	Decrease/No Change of PD-L1 CTCs	Increase of PD-L1^−^ CTCs
Responding patients	10 (decrease), 11 (no change)	0
Non-responding patients	0	17

**Table 2:** Univariate and Multivariate Cox regression analysis.

Variable		Univariate Analysis			Multivariate Analysis		
		Hazard Ratio	95% CI	*p* Value	Hazard Ratio	95% CI	*p* Value
		All mCRC (*n* = 40)					
PD-L1+ CTCs	Positive vs. Negative	16.541	7.311–38.414	<0.001	0.09	0.01–0.751	0.016
ECOG PS	≥1 vs. 0	1.444	0.511–3.121	0.481			
KRAS	Mutant vs. WT	20.7	9.474–45.415	<0.001	3.051	1.121–7.751	0.030
BRAF	Mutant vs. WT	0.399	0.210–0.851	0.004			
PLR	≥200 vs. <200	0.21	0.041–0.815	<0.001	0.345	0.131–0.845	0.018
N sites metastases	≥2 vs. 1	4.334	2.580–7.280	<0.001			

Abbreviation: mCRC, metastatic colorectal cancer; CI, confidence interval; CTCs, circulating tumour cells; ECOG PS, Eastern Cooperative Oncology Group Performance status; PLR, platelet-to-lymphocyte ratio; WT, wild type.

## Abbreviations

mCRC	Metastatic Colorectal cancer
CTCs	Circulating Tumour Cells
PD-L1	Programmed Death-Ligand 1
PLR	Platelet-to-lymphocyte ratio
ECOG PS	Eastern Cooperative Oncology Group Performance Status
PFS	Progression-free survival
OS	Overall Survival
DAPI	40,6-diamidino-2-phenylindole

## Appendix A

**Table A1:** Clinicopathological characteristics of all 40 patients enrolled.

Variable	No. of Patients (%)
Age (median = 65)	
<65	12 (30)
≥65	28 (70)
Gender	
M	25 (63)
F	15 (37)
ECOG PS	
0	29 (73)
1–2	11 (27)
Primary tumour Location	
Colon	39 (98)
Rectum	1 (2)
KRAS status	
WT	13 (35)
Mutated	26 (65)
BRAF status	
WT	39 (98)
V600E	1 (2)
Metastatic sites	
Liver	35 (88)
Lymph Nodes	33 (83)
Lungs	8 (20)
Others	12 (30)
N° of metastatic sites	
1	6 (15)
≥2	34 (85)
PLR	
Mean (SD)	212.5 (208.32)
<200	24 (60)
≥200	16 (40)

## Appendix B

**Table A2:** Patients’ number of CTCs and percentage of PDL1+ CTCs.

Patients	Number of CTCs	% of PD-L1+CTCs
P1	12	20
P2	50	30
P3	60	10
P4	21	5
P5	22	80
P6	135	50
P7	110	40
P8	210	94
P9	40	81
P10	35	88
P11	70	16
P12	190	70
P13	50	91
P14	110	16
P15	410	60
P16	13	21
P17	60	30
P18	45	12
P19	25	6
P20	100	78
P21	135	40
P22	140	40
P23	220	81
P24	85	70
P25	70	0
P26	70	0
P27	10	0
P28	10	0
P29	20	0
P30	12	0
P31	41	0
P32	18	0
P33	22	0
P34	110	0
P35	24	0
P36	46	0
P37	25	0
P38	17	0
